# Drug-sensitive *FGFR3* mutations in lung adenocarcinoma

**DOI:** 10.1093/annonc/mdw636

**Published:** 2016-12-19

**Authors:** P. Chandrani, K. Prabhash, R. Prasad, V. Sethunath, M. Ranjan, P. Iyer, J. Aich, H. Dhamne, D. N. Iyer, P. Upadhyay, B. Mohanty, P. Chandna, R. Kumar, A. Joshi, V. Noronha, V. Patil, A. Ramaswamy, A. Karpe, R. Thorat, P. Chaudhari, A. Ingle, A. Choughule, A. Dutt

**Affiliations:** 1 Integrated Genomics Laboratory, Advanced Centre for Treatment, Research and Education in Cancer, Tata Memorial Centre, Navi Mumbai;; 2 Homi Bhabha National Institute, Training School Complex, Anushakti Nagar, Mumbai;; 3 Department of Medical Oncology, Tata Memorial Hospital;; 4 Small Animal Imaging Facility, Advanced Centre for Treatment, Research and Education in Cancer, Tata Memorial Centre, Navi Mumbai;; 5 AceProbe Technologies Pvt. Ltd, New Delhi, India;; 6 Department of Pathology, Tata Memorial Hospital;; 7 Laboratory Animal Facility, Advanced Centre for Treatment, Research and Education in Cancer, Tata Memorial Centre, Navi Mumbai

**Keywords:** lung adenocarcinoma, actionable mutations, fibroblast growth factor receptor 3, oncogene, FGFR inhibitors, mass spectrometry

## Abstract

**Background:**

Lung cancer is the leading cause of cancer-related deaths across the world. In this study, we present therapeutically relevant genetic alterations in lung adenocarcinoma of Indian origin.

**Materials and methods:**

Forty-five primary lung adenocarcinoma tumors were sequenced for 676 amplicons using RainDance cancer panel at an average coverage of 1500 × (reads per million mapped reads). To validate the findings, 49 mutations across 23 genes were genotyped in an additional set of 363 primary lung adenocarcinoma tumors using mass spectrometry. NIH/3T3 cells over expressing mutant and wild-type *FGFR3* constructs were characterized for anchorage independent growth, constitutive activation, tumor formation and sensitivity to FGFR inhibitors using *in vitro* and xenograft mouse models.

**Results:**

We present the first spectrum of actionable alterations in lung adenocarcinoma tumors of Indian origin, and shows that mutations of *FGFR3* are present in 20 of 363 (5.5%) patients. These *FGFR3* mutations are constitutively active and oncogenic when ectopically expressed in NIH/3T3 cells and using a xenograft model in NOD/SCID mice. Inhibition of *FGFR3* kinase activity inhibits transformation of NIH/3T3 overexpressing *FGFR3* constructs and growth of tumors driven by *FGFR3* in the xenograft models. The reduction in tumor size in the mouse is paralleled by a reduction in the amounts of phospho-ERK, validating the *in vitro* findings. Interestingly, the *FGFR3* mutations are significantly higher in a proportion of younger patients and show a trend toward better overall survival, compared with patients lacking actionable alterations or those harboring *KRAS* mutations.

**Conclusion:**

We present the first actionable mutation spectrum in Indian lung cancer genome. These findings implicate *FGFR3* as a novel therapeutic in lung adenocarcinoma.

## Introduction

Lung cancer is the leading cause of cancer-related deaths worldwide, accounting for over a million deaths annually [1]. Molecularly targeted therapies improve outcome for lung adenocarcinoma patients whose tumors harbor mutant *EGFR* or translocated *ALK*, *RET* or *ROS1*, with an encouraging response for those with mutated *BRAF*, *MET*, *NTRK-1 & 2* and *ERBB2* [2–5]. Such oncogenic somatic alterations though vary across populations/ethnic groups, e.g. *EGFR* mutations are present in over 30% of East Asian lung adenocarcinoma patients, however, they are only found in ∼23%–25% of Indian and 10% of Western lung adenocarcinoma patients [6–8]. Similarly, *KRAS* mutations are present at 60% lower frequency in Indian lung adenocarcinoma patients than compared with the Caucasian population [3, 9, 10]. The diversity in somatic alterations lends similarity to the known plurality in clinical response based on ethnicity and divergent genetic and environmental factors [11], Thus, besides the unmet need for additional therapeutic targets in lung adenocarcinoma patients, it is equally pertinent to profile known oncogenic somatic alterations across different populations to understand their landscape of variability.

Here, in an attempt to profile for activating alterations, we have generated a comprehensive mutational spectrum of activating alterations prevalent among lung adenocarcinoma patients of Indian origin, considered to be an admixture population of non-European Caucasian and Ancestral South Indians. We also report the first incidence of activating and drug sensitive *FGFR3* mutations in lung adenocarcinoma. *FGFR3* mutated samples, with ∼5% population frequency, form a distinct subclass apart from *EGFR*, *KRAS* and *EML4-ALK*.

## Materials and methods

### Patients

To profile for therapeutically relevant genome alterations in lung adenocarcinoma of Indian origin, FFPE blocks with known *EGFR* mutation status for 45 consecutive histologically confirmed lung adenocarcinoma patients tumor samples (stage IV, 49% males and 51% non-smokers) for sequencing and an additional set of 363 consecutive lung adenocarcinoma patients tumor samples (stage IV, 62% males and 73% non-smokers) for mass spectrometry were retrospectively collected from Tata Memorial Hospital (supplementary Table S1, available at *Annals of Oncology* online).

### Pooling of samples, target gene-capturing and next generation sequencing

A set of 45 lung adenocarcinoma samples were sequenced using pooled sequencing approach to capture low-frequency variants [12–14]. Briefly, 45 samples were divided into duplicate pools of different population sizes (supplementary Figure S1, available at *Annals of Oncology* online), i.e. 2 pools of 5 individuals (5XA and 5XB), 2 pools of 10 individuals (10XA and 10XB) and 1 pool of 15 individuals (15X) for next-generation sequencing (NGS) of 676 genomic regions of 158 genes as described earlier [15].

### Discovery of genomic variants using computational analysis

FASTQ files were analyzed using BWA-Picard-GATK/MuTect pipeline generating 3349 unique variants (supplementary Table S2, available at *Annals of Oncology* online). Polymorphisms overlapping with dbSNP database (v.142) and Indian specific SNP database TMC-SNPdb derived from whole exome sequencing of 62 normal samples [16] were filtered (supplementary Figures S2 and S3, available at *Annals of Oncology* online). Stringent mutation analysis was carried out as further detailed in supplementary methods, available at *Annals of Oncology* online to derive list of significant mutations for further validation (supplementary Tables S2 and S3, available at *Annals of Oncology* online).

### Mass spectrometry based genotyping

Briefly, PCR and extension primers for 49 mutations in 23 genes were designed using single base extension based mass spectrometry assay design 3.1 software (supplementary Table S4, available at *Annals of Oncology* online). Mutation calls were analyzed using Typer 4 (Sequenom, Inc., USA) and were reviewed by manually observing mass spectra.

### Cell culture, anchorage-independent growth assay and immunoblotting

NIH/3T3 cells transduced with *FGFR3* wild-type and mutant construct were used for induction and drug inhibition study as detailed in supplementary methods, available at *Annals of Oncology* online. Anchorage independent growth assay and immunoblotting were carried out as described earlier [17], and as detailed in the supplementary methods, available at *Annals of Oncology* online.

### Xenograft development

A cohort of eight NOD-SCID or nude mice per clone were subcutaneously injected with five million cells for tumor formation in 2–3 months. Inhibitor BGJ-398 [18] was given at 15 and 30 mg/kg along with vehicle control (10% Tween-80) independently to randomized xenograft groups after tumor size reaching ∼150 mm^3^. Tumor size was measured every alternate day using a Vernier caliper during the 14 day period of drug treatment. microPET-CT scan was carried out at the end of the drug treatment.

### Immunohistochemistry

Immunohistochemistry analysis was carried out as described earlier [19] and detailed in supplementary methods, available at *Annals of Oncology* online.

### Overall survival analysis

Overall survival (OS) of patients was assessed using Kaplan–Meier method using R and IBM SPSS software package, as detailed in supplementary methods, available at *Annals of Oncology* online. The end point was taken as date of death with censoring implied at the date of the last contact.

## Results

### Recurrent *FGFR3* mutations in lung adenocarcinoma patient of Indian origin

**Figure 1.**
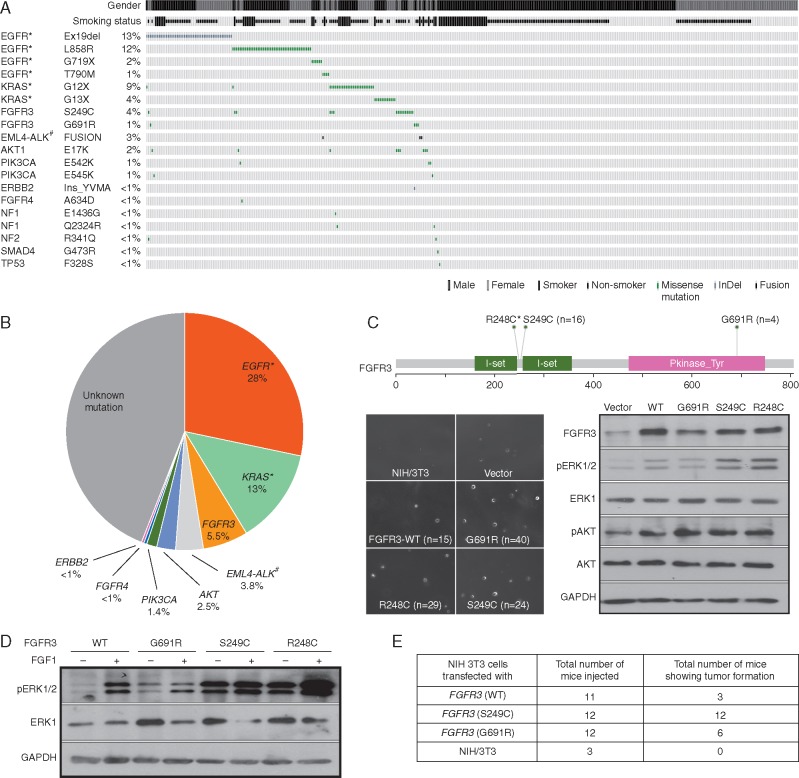
Recurrently mutated genes in lung adenocarcinoma. (A) Validated mutations in 363 samples identified by single base extension-based mass spectrometry were visualized using OncoPrinter tool available at cBioPortal. The asterisk (*) denotes genes genotyped using TaqMan and SNaPShot assays in addition to the mass spectrometry. # Fusion frequency was determined using fluorescent in-situ hybridization in 79 of 363 patients. (B) Pie-chart representation of the frequency of clinically relevant genes observed in 363 lung adenocarcinoma patients of Indian origin. (C) Upper panel: Schematic diagram to represent position of point mutations identified in *FGFR3* using next-generation sequencing. Numbers of patients found to be mutated by mass-spectrometry-based genotyping are denoted in brackets. Lower left panel: Representative pictures and soft agar colony count (averaged from triplicate) are shown for NIH/3T3 clones. Lower right panel: immunoblot analysis of NIH/3T3 clones using anti- FGFR3, total- and phospho- ERK1/2 and AKT antibody. (D) Immunoblot analysis of NIH/3T3 clones with (50 ng/ml) and without FGF1 ligand treatment of total- and phospho-ERK1/2. GAPDH was used as loading control in immunoblots. (E) *In vivo* tumorigenicity of NIH/3T3 cells expressing *FGFR3* mutants and wild-type in NOD-SCID mice is shown. Detailed figure legend can be found in supplementary materials, available at *Annals of Oncology* online.

To identify low frequency and ethnic-specific therapeutically relevant genome alterations in lung adenocarcinoma of Indian origin, we sequenced 45 primary lung adenocarcinoma stage IV tumors (supplementary Table S1, available at *Annals of Oncology* online) for 676 amplicons at an average coverage of 1500× (reads per million mapped reads), as described in supplementary Figures S1–S5 and Tables S2, S3, available at *Annals of Oncology* online. To validate the findings, we selected 49 mutations occurring across 23 genes based on their recurrence and therapeutic significance (supplementary Table S4, available at *Annals of Oncology* online), for genotyping in an additional set of 363 primary lung adenocarcinoma stage IV tumors (Figure 1A; supplementary Table S5, available at *Annals of Oncology* online) using mass spectrometry.

Based on the mutation profiling of 363 lung adenocarcinoma patients, we present the first portrait of activating mutations present in the Indian lung cancer genome (Figure 1B), wherein 160 of 363 patients were found to harbor activating mutations across 8 genes at following frequency: *EGFR* (28.4%), *KRAS* (13%), *ALK* (3.8%), *AKT1* (2.5%), *PIK3CA* (1.4%), *FGFR4* (0.4%) and *ERBB2* (0.3%) as shown in Figure 1A, consistent with earlier reports [6, 8, 9]. In addition, 3 of 79 patients were found to harbor *EML4-ALK* translocation as determined by FISH. Among the other most significantly mutated genes, we found recurrent *FGFR3* mutations in 20 of 363 tumors (5.5%), of which 7 co-occurred in samples harboring *EGFR* (*n = 5*) and *KRAS* (*n = 2*) mutations. In total, 16 patients harbored *FGFR3* (S249C) mutation; and 4 patients harbored a novel *FGFR3* (G691R) mutation (Figure 1A and C, upper panel; supplementary Figure S6 and Table S6, available at *Annals of Oncology* online). Interestingly, *FGFR3* (S249C) mutation has previously been described as activating and drug sensitive in lung squamous [20], while the novel *FGFR3* (G691R) mutation was predicted to be deleterious based on using 7 of 10 functional prediction tools (supplementary Table S3, available at *Annals of Oncology* online).

### *FGFR3* mutations in lung adenocarcinoma are activating *invitro* and *invivo*

To test whether the recurrent *FGFR3* mutations found in this study are activating we transduced NIH/3T3 fibroblast cells with retroviruses encoding the *FGFR3* G691R mutation along with WT *FGFR3* and the previously characterized *FGFR3* (R248C) and (S249C) mutations [20]. Similar to *FGFR3* R248C and S249C, the ectopic expression of the novel G691R mutant clone in pooled NIH/3T3 cells conferred anchorage-independent growth, forming threefold more colonies in soft agar than cells expressing WT *FGFR3* (Figure 1C, left panel), despite higher expression levels of WT *FGFR3* (Figure 1C, right panel). The transformation was accompanied by elevated phosphorylation of the downstream ERK1/2 and AKT1 in a constitutive manner (Figure 1D). Furthermore, consistent with the *in vitro* data, NIH/3T3 cells expressing transforming *FGFR3* mutations or WT when injected subcutaneously into NOD/SCID mice formed tumors within 2 months post injection of cells. While 3 of 11 mice injected with cells expressing *FGFR3* WT formed tumors, 12 of 12 mice injected with cells expressing *FGFR3* S249C; and 6 of 12 mice injected with cells expressing *FGFR3* G691R formed tumors (Figure 1E). The tumor size doubling time was ∼7 days for cells expressing *FGFR3* (G691R), ∼5 days for cells expressing *FGFR3* (S249C); the *FGFR3*-WT tumors doubled in size in ∼9–10 days.

### *FGFR3* mutations in lung adenocarcinoma are sensitive to inhibitors *invitro* and *invivo*

**Figure 2.**
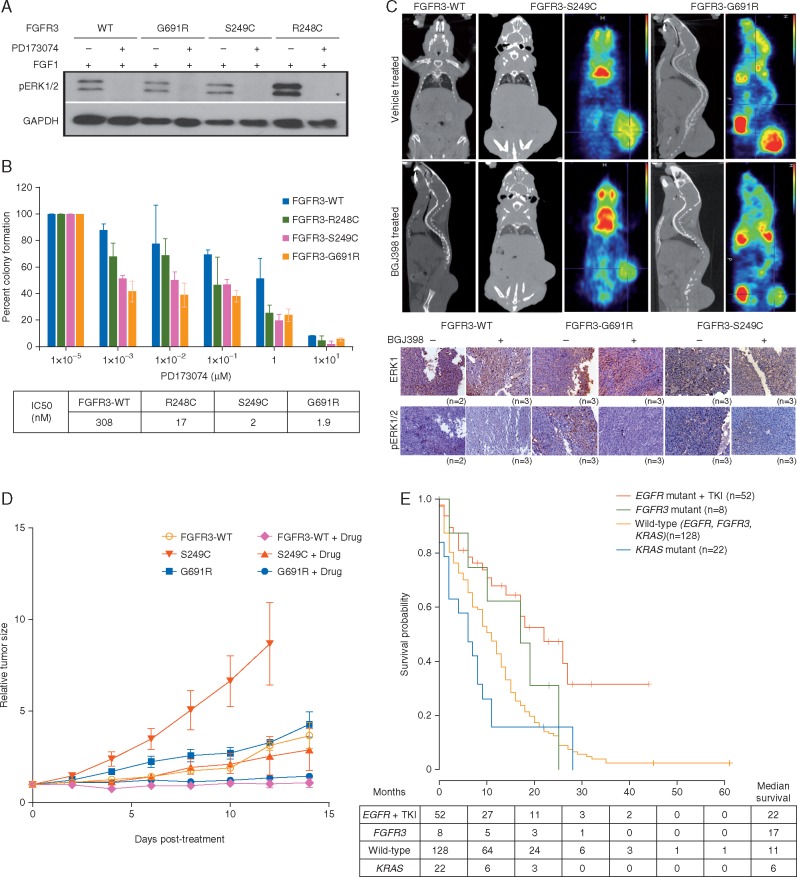
Transformed NIH/3T3 cells and xenografts are sensitive to FGFR inhibitor. (A) Immunoblot analysis of NIH/3T3 clones treated with FGF1 (50 ng/ml) followed by FGFR inhibitor PD173074 (2 µM) is shown. GAPDH was used as loading control. (B) Soft agar colony count (averaged from 3 replicates) and IC-50 values of NIH/3T3 clones expressing wild-type or mutant *FGFR3*, treated with increasing concentration of PD173074 is shown. (C) Upper panel: NIH/3T3 xenografts developed into NOD-SCID mice were treated with FGFR inhibitor BGJ-398 or vehicle for 21 days. CT-scan and a readout for relative ^18^F-FDG uptake is shown by a gradient color code with red indicating as maximum uptake. Lower panel: Immuno-histochemical staining of total and phospho-ERK1/2 is shown in xenografts treated with drug and vehicles. (D) The plot shows tumor size (normalized to the size at day 0 of drug treatment) during the course of drug treatment indicating a reduced tumor size in drug-treated xenografts. (E) Clinical follow-up of total 205 patients for up to 62 months was used for the Kaplan–Meier analysis. *EGFR* positive patients received Gefitinib as a regular therapeutic regimen while rest of the patients received conventional chemotherapy. The table below the plot indicates patients at risk during the course of 60 months and median survival for each mutant group. Detailed figure legend can be found in supplementary materials, available at *Annals of Oncology* online.

We further show that inhibition of FGFR3 kinase activity using pan FGFR inhibitor PD173074 block the constitutive phosphorylation of ERK1/2 (Figure 2A). Similarly, the treatment of cells harboring activating *FGFR3* mutations with PD173074 result in a marked decrease in colony formation in soft agar and cell survival in liquid culture (Figure 2B). Extending the effect *in vivo* studies, when tumors reached ∼100–200 mm^3^ in all mice injected with NIH/3T3 cells began treatment with 15 or 30 mg/kg BGJ398—a selective FGFR inhibitor currently under clinical trials for various cancer types (as PD173074 is incompatible with *in vivo* studies [21]), or vehicle for 14 days. Tumors treated with BGJ398 slowed or reversed their growth compared with vehicle (Figure 2C, upper panel), so that by the end of the study, the effect on tumor burden in vehicle-treated versus BGJ398-treated mice were 3.3-folds in *FGFR3* (S249C), three-fold in *FGFR3* (G691R) and 2.25-fold in *FGFR3*-WT xenografts (Figure 2D). This reduction in tumor size was paralleled by a reduction in the amounts of phospho-ERK1/2 in immuno-histochemical analyses (Figure 2C, lower panel) of explanted tumors, validating our *in vitro* findings that MAPK signaling is the key pathway engaged by mutated *FGFR3*.

### Correlation of *FGFR3* mutations with clinicopathological features of lung cancer patients

Clinically, lung adenocarcinoma patients with *FGFR3* mutation positive tumors expressing higher activated MAPK levels (supplementary Figure S7, available at *Annals of Oncology* online) show a better trend in OS with 17 months (*n= 8*; 95% CI: 6.4–27.5; HR: 0.6) compared with 14 months (*n= 197*; 95% CI: 8.7–13.2) in patients with wild-type *FGFR3* (Figure 2E). The OS trend in lung adenocarcinoma patients though is similar to bladder urothelial carcinomas and skin cutaneous melanoma patients, but not to head and neck cancer and lung squamous carcinoma patients, based on our analysis using cBioPortal for survival of patients harboring activating *FGFR3* mutations in different cancers (supplementary Figure S8, available at *Annals of Oncology* online). Furthermore, the *FGFR3* mutations were observed to be significantly higher in patients <45 years (9 of 95) than in patients >45 years (11 of 269) (*P = 0.048*) but not with their gender and smoking status (supplementary Table S7, available at *Annals of Oncology* online). The sample size in this study, however, is underpowered to reach statistical significance for survival data.

## Discussion

We present the first portrait of clinically actionable alterations in lung adenocarcinoma of Indian origin that includes *EGFR*, *KRAS*, *EML4-ALK*, *AKT1*, *PIK3CA*, *FGFR4* and *ERBB2*, similar to that identified in other ethnic groups [5, 22, 23], and an additional subset of patients with *FGFR3* mutations. Ethnic-specific variations have been well known in lung cancer [24, 25] across different populations. We observed 28.4% *EGFR* mutations and 13% *KRAS* mutations in lung adenocarcinoma patients, consistent with our previous report [6, 9]. Similarly, variation in frequency of other molecular alterations is also observed such as 3% *EML4-ALK* alteration in our study compared with 8% in Caucasian population [3] and in 5% Chinese population [23]. *ERBB2* mutation found at <1% frequency in our cohort exists at ∼2%–3% among the Caucasian [3] and Chinese populations [23]. Similarly, *AKT1* mutations were found at higher than the reported <1% in both Caucasian [3] and Chinese populations [23] indicating the higher therapeutic relevance of *AKT1* targeted compounds in Indian population.

We have also identified frequent and recurrent drug sensitive *FGFR3* mutations in lung adenocarcinoma patients. Among the Caucasians, activating mutations in *FGFR3* have been earlier reported in bladder carcinoma [26], lung squamous cell carcinomas [20] and cervical cancer [27], but were found to be largely absent in lung adenocarcinomas [23, 28, 29], except for Imielinski et al. who reported non-recurrent somatic *FGFR3* mutations of unknown functional significance in 3 of 183 lung adenocarcinoma patients [10]. On the other hand, the presence of frequent *FGFR3* mutations (with unknown driving potential) is tangentially referred to in the literature among Korean lung adenocarcinomas patients [30]. Along with these reports, our finding of activating *FGFR3* mutations in lung adenocarcinoma patients provides an interesting convergence with mouse genetic experiments wherein activated FGF9-FGFR3 signal acts as the primary oncogenic pathway involved in initiation of lung adenocarcinoma [31, 32].

Analyzing the potential effect of *FGFR3* driver mutations on survival of lung cancer patients, we observed a trend toward better survival for *FGFR3* mutations in lung adenocarcinoma, compared with lung adenocarcinoma patients with wild-type *FGFR3* and those harboring *KRAS* mutation, similar to as reported in the bladder and skin cancer [33]. Thus, *FGFR3* mutation represents an opportunity for targeted therapy in lung adenocarcinoma. FGFR inhibitors, which are currently in clinical testing in tumor types bearing genetic alterations in FGFR genes [34, 35], may be extended to evaluate in patients with *FGFR3-*mutated lung adenocarcinoma. Finally, with a broader emerging role across different cancers [20, 36–39], this study further underscores that *FGFR* family may potentially join the *EGFR* family as a widespread target for therapeutic intervention in several human cancers.

## Supplementary Material

Supplementary Data
