# Supplementary material for: Drug-sensitive FGFR3 mutations in lung adenocarcinoma
Source: Ann Oncol. 2016 Dec 19;28(3):597–603. doi: 10.1093/annonc/mdw636 (PMC5391708; doi:10.1093/annonc/mdw636)
Supplement: Supplementary Data [file mdw636_supp.zip › Chandrani et al. suppl Methods and Figures 20161110.pdf]

Supplementary Methods

Sample processing

To profile for therapeutically relevant genome alterations in lung adenocarcinoma of Indian origin, FFPE blocks for 45 consecutive histologically confirmed lung adenocarcinoma patients tumor sample for sequencing and an additional set of 363 consecutive lung adenocarcinoma patients tumor sample for mass spectrometry were retrospectively collected from Tata Memorial Hospital, where an adequate amount of acceptable quality of genomic DNA was available (Supplementary Table S1). The Institutional Review Board (IRB) and the Ethics Committee (EC) of Tata Memorial Center (TMC)- Advanced Centre for Treatment, Research and Education in Cancer (ACTREC) (Mumbai, India) approved the project (# 55 and 108) during the 21st TMC-ACTREC IRB meeting. The patient characteristics including the age, gender, smoking/tobacco use, and histopathology were recorded.

Pooling of samples, target gene-capturing and next generation sequencing

Total, 45 samples were divided into duplicate pools of different population size (Supplementary Figure-S1) i.e. 2 pools of 5 individuals (5XA and 5XB), 2 pools of 10 individuals (10XA and 10XB), and, 1 pool of 15 individuals (15X). All 5 pools were submitted to RainDance Technologies Inc., USA to prepare next-generation sequencing (NGS) libraries capturing 676 genomic regions of 158 genes (127 KB of DNA) using RainDance Cancer panel, as described earlier [1]. The NGS libraries were then submitted to Sandor LifeSciences Pvt. Ltd. (Hyderabad, India) for sequencing using paired-end chemistry using two lanes of Illumina flow cells on GA-IIx giving expected sequencing coverage of more than 1,500X per base.

Discovery of genomic variants using computational analysis

FASTQ files for each pool were fed into mapping/alignment program BWA for mapping onto reference human genome sequence GRCh37. Variants were called using GATK [2] and Mutect [3] to generate median 837 coding variants per pool (range: 756 – 2145) representing total 3349 unique variants (Supplementary Table S2). Mutational signature of FFPE tissues [4] (C:G/T:A mutations) and polymorphisms overlapping

with dbSNP database (v.142) and TMC-SNPdb database (Indian ethnicity specific SNP database) [5] (Supplementary Figure S2, S3) were filtered out. We also used 9 functional prediction tools through dbNSFP to further prioritize cancer related variants called as deleterious by at-least 7 tools (Supplementary Table S3).

Genotyping using single base extension based mass spectrometry

DNA from 363 samples was submitted to AceProbe technologies for mass spectrometry following companies standard protocol. Briefly, PCR and extension primers for 49 mutations in 23 genes were designed using single base extension based mass spectrometry assay design 3.1 software (Supplementary Table S4). Mutation calls were analysed using Typer 4 (Sequenom Inc., USA) and were reviewed by manually observing mass-spectra.

Cell culture, reagents, transfection, and infection

Pre- authenticated NIH/3T3 cells were obtained from ATCC (CRL-1658) and used within 6 months of thawing. *FGFR3* was cloned into pBABE-puro from pDONR223 (was a gift from William Hahn & David Root addgene plasmid # 23933) for retroviral production [6]. *FGFR3* mutants were generated by site-directed mutagenesis using the Quikchange II kit (cat.no. 200523) and confirmed by Sanger sequencing. NIH/3T3 cells were infected with retroviruses in the presence of 8 µg/ml polybrene. Infected cells were puromycin (Sigma) selected (2 µg/ml) after two days of viral transduction. To induce cells with FGF1, cells were placed in media containing 0.5% calf serum 12 hours before 50 ng/ml FGF1 (Abcam ab91374) stimulation for 20 min at 37°C. PD173074 was purchased from Calbiochem and diluted in DMSO to the indicated concentrations. BGJ398 was purchased from Santa Cruz biotechnology and diluted in 10% tween-80.

Anchorage-independent growth assay

Anchorage independent growth assay was performed as described earlier [7]. Briefly, 3 independent sets of 5 x 10^3 and 20 x 10^3 cells were suspended in a layer of 0.4% select agar (Gibco/Invitrogen) and plated on a bottom layer of 0.8% select agar with

PD173074 added at described concentration. IC_{50} was determined by nonlinear regression with Prism GraphPad software and Dr Fit tool.

Immunoblotting

Immunoblots were prepared as described earleir [7]. Primary antibodies used for immunoblotting were: anti-FGFR3 (Santa Cruz Biotechnology; 1:500), anti- total-ERK1/2 (Santacruz Biotechnology; 1:200), phospho-ERK1/2 (Cell signaling Technology; 1:1000). Secondary antibodies used were- Goat anti-rabbit (Santacruz Biotechnology; 1:2000) and Bovine anti-mouse (Santacruz Biotechnology; 1:2000). Pierce ECL (Thermo Scientific, USA) substrate was used for visualizing the blots.

Xenograft development

The study was approved by the Institutional Animal Ethics Committee of ACTREC, Navi Mumbai. For this study 8-weeks old male NOD SCID mice were procured from the Animal Facility of ACTREC, Navi Mumbai. A cohort of 8 NOD-SCID mice per clone were subcutaneously injected with 5 million cells for tumor formation in 2-3 months. Tumors were collected to subcutaneously graft ~2-3 mm tumor piece in further expanded a set of mice. Inhibitor BGJ-398 [8-10] was given at 15 and 30 mg/kg along with vehicle control (10% tween-80) independently to randomised xenograft groups after tumor size reaching ~150 mm^3. Tumor size was measured every alternate day using Vernier calliper and microPET-CT scan during 14 days' drug treatment.

Tissue processing and Immunohistochemistry

Immunohistochemistry analysis was performed as described earlier [11] Briefly, FFPE blocks were sectioned at 4µm, baked at 58 ^oC for 30 minutes before staining, followed by deparaffinization, rehydration, and quenching. Tissue sections were incubated overnight at room temperature with anti- total-ERK1/2 (Santacruz Biotechnology) and phospho-ERK1/2 (Cell signaling Technology) in PBS. Further, sections were counter stained with haematoxylin and covered with DPX mounting reagent and cover slip. Imaging was performed at 10X & 20X magnification using an upright microscope.

Overall survival analysis

We used R packages survival (http://cran.r-project.org/package=survival), survMisc (https://cran.r-project.org/web/packages/survMisc/index.html) and IBM SPSS software for Kaplan-Meier estimation of patient survival. Overall survival of patients from TCGA cohort was estimated by querying cBioPortal (http://www.cbioportal.org/) for cancer types indicated.

Expanded Figure legends

Figure-1: Recurrently mutated genes in lung adenocarcinoma. (A) Validated mutations in 363 samples identified by single base extension based mass spectrometry are visualized using OncoPrinter tool available at cBioPortal. The cases were sorted by gene alteration frequency and grouped by mutation types. Grey bar indicates each patient negative for respective mutation and or annotation in the rows. Missense mutations (green), small deletions (brown), and fusion events (black) are indicated for respective genes/patients. Smoking status (smokers: black box, non-smokers: half black box, grey: information not available), gender (male: black outline, female: dark grey outline) are indicated in top annotation track wherein light grey box represents unavailability of data. The asterisk (*) denotes that genes genotyped using TaqMan and SNaPShot assays in addition to single base extension based mass spectrometry. # Fusion frequency was determined using fluorescent in-situ hybridization in only 79 patients out of 363 total. (B) Pie-chart representation of the frequency of clinically relevant genes observed in 363 Indian lung adenocarcinoma. (C) Upper panel: Schematic diagram of two ligand binding domain and one kinase domain point mutations identified in *FGFR3* using next-generation sequencing analysis. Number of patients found to be mutated by mass-spectrometry based genotyping are denoted in brackets. Asterisk indicates the mutations found in next-generation analysis but was not found in genotyping assay. Lower left panel: NIH/3T3 cells stably expressing wild-type or mutant *FGFR3*, as indicated, were suspended in soft agar for colony formation assay and photographed after 3 weeks of incubation at 37°C. Representative pictures and colony count (averaged from triplicate) are shown for NIH/3T3 clones as indicated. Lower right panel: Immunoblot analysis of NIH/3T3 clones for anti- FGFR3, total- and phospho- ERK1/2 and AKT. GAPDH was used as loading control. (D) Immunoblot analysis of NIH/3T3 clones with and without ligand (50 ng/ml FGF1) treatment for total- and phospho- ERK1/2 demonstrating ligand-independent activation of signaling pathway. GAPDH was used as loading control. (E) *In-vivo* tumorigenicity of NIH 3T3 cells expressing *FGFR3* mutants and wild-type is shown. NIH/3T3 cells expressing *FGFR3* constructs were subcutaneously injected into NOD-SCID mice for tumor formation to be observed in 2 months.

Figure-2: Transformed NIH/3T3 cells and xenografts are sensitive to FGFR inhibitor. (A) Immunoblot analysis of NIH/3T3 clones treated with FGF1 (50 ng//ml) followed by FGFR inhibitor PD173074 (2µM) is shown indicating blockage of signaling pathway (phospho-ERK1/2) in drug-treated cells. GAPDH was used as loading control. (B) NIH/3T3 clones expressing wild-type or mutant *FGFR3*, as indicated, were suspended in soft-agar plates with increasing concentration of PD173074. Quantification was averaged from 3 replicates. IC-50 calculations were carried out by using non-linear regression in GraphPad software. (C) Upper panel: NIH/3T3 clones were subcutaneously injected into NOD-SCID for tumor formation in ~2 months. Selective FGFR inhibitor BGJ-398 or vehicle treatment was administered orally in mice after tumor size reaching ~100-200 mm^3. ^{18}F-FDG uptake was studied in these tumors after 21 days of drug treatment. A readout for relative ^{18}F-FDG uptake is shown by a gradient color code with red indicating as maximum uptake. (C) Lower panel: Immuno-histochemical staining of total- and phospho- ERK1/2 is shown in xenografts treated with drug and vehicles. (D) Tumor size was also measured every alternate day using Vernier calliper in each xenograft of treatment and control arm. The plot shows tumor size (normalized to the size at day 0 of drug treatment) during the course of drug treatment indicating a reduced tumor size in drug-treated mice. (E) Clinical follow-up of total 205 patients for up to 62 months was used for Kaplan-Meier analysis. *EGFR* positive patients received Gefitinib as a regular therapeutic regimen while rest of the patients were received conventional chemotherapy. The table below the plot indicates patients at risk during the course of 60 months and median survival for each mutant groups. Median OS of patients with *FGFR3* mutant NSCLC was 17 months (green; n= 8; 95% CI: 6.4-27.5; HR: 0.6), *EGFR* mutation having received TKI was 22 months (red; n= 53; 95% CI: 18.4-30.3; HR: 0.4), *KRAS* mutation was 6 months (blue; n= 22; 95% CI: 5.1-18.9; HR: 1.3) while *EGFR*, *KRAS* and *FGFR3* mutation negative patients was 11 months (black; 95% CI: 8.7-13.2).

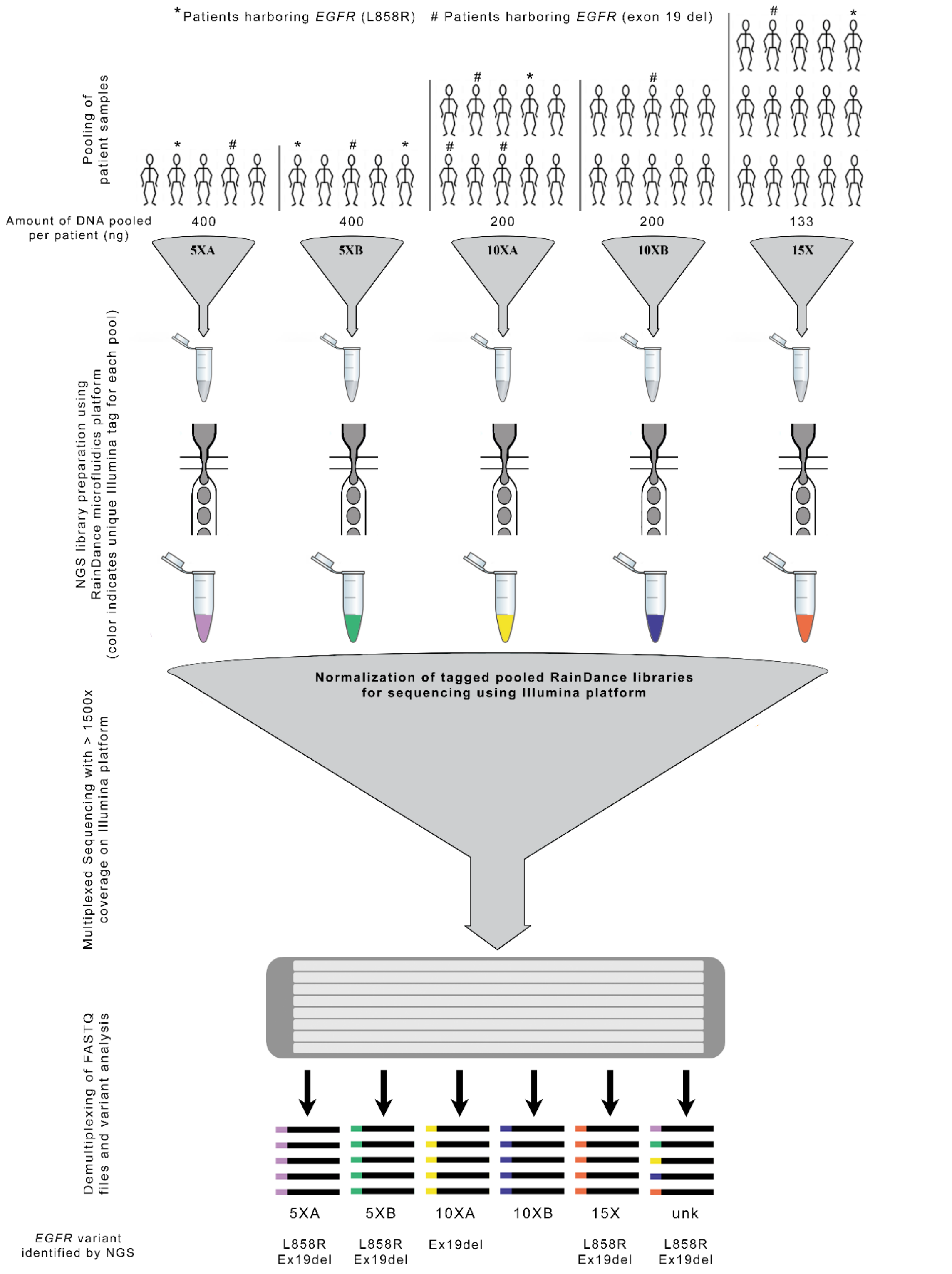

Supplementary Figure-S1: Schematic diagram of pooled next-generation sequencing of 45 lung adenocarcinomas. (A) Set of 45 lung adenocarcinoma sample with known *EGFR* mutation status were divided into duplicate pools of different population size i.e. 2 pools of 5 individuals (5XA and 5XB), 2 pools of 10 individuals (10XA and 10XB), and, 1 pool of 15 individuals (15X). 400 ng of each sample in 5X

pool; 200 ng of each samples in the 10X pool and 133 ng of each sample in 15X to make the respective pools. All 5 pools were used to capture 676 genomic regions of 158 genes using RainDance Cancer panel that was unique tagged as shown. The tagged libraries were normalized and sequenced on Illumina GAIIx. The multiplexed sequencing data was de-multiplexed per the unique tags into five fastq files, each corresponding to five pools. Reads with unidentifiable or degenerate barcode sequences were put together in pool of unknown size labeled as “unk”. The ability to make variant calls for *EGFR* within each pool served as the positive control that was found to be largely concordant.

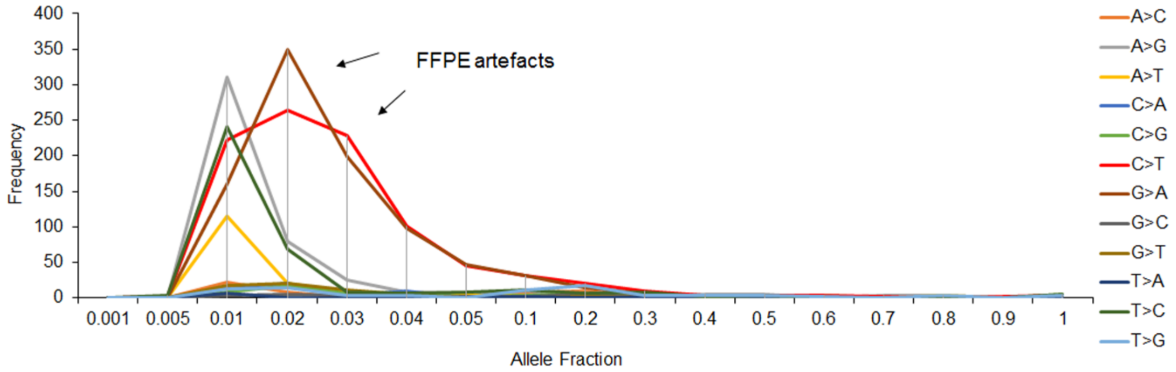

Supplementary Figure-S2: Allele fraction distribution in high-throughput sequencing data. All the variants are shown by a base-pair change at various allele fractions. Arrow points to C:G/T:A variants, known as a product of FFPE artifacts, to be enriched below 0.05 allele fraction which was filtered out from the study.

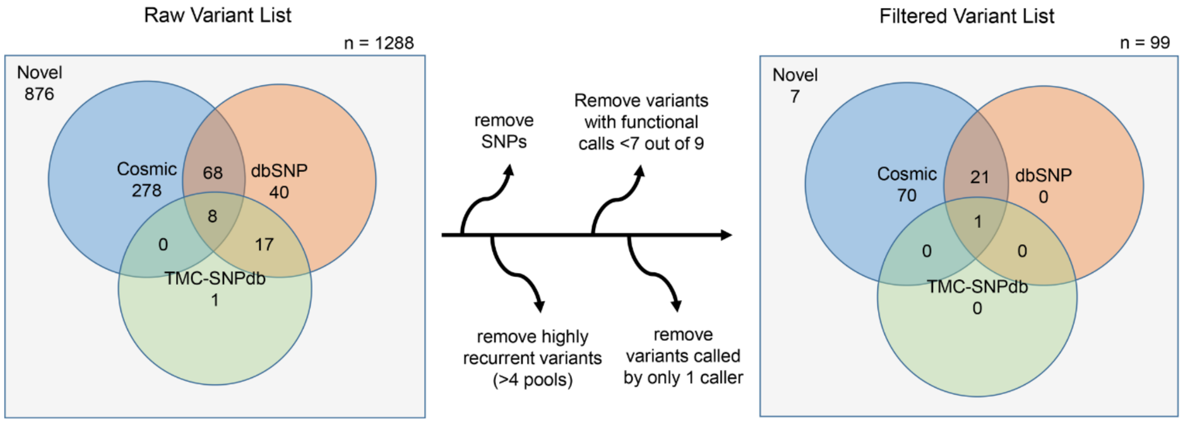

Supplementary Figure-S3: Variant prioritization strategy to enrich cancer-related variants. The box on left shows total coding variants called in the cohort. The box on the right shows filtered variants after removal of known SNPs, highly recurrent

variants (>4 pools), variants failing to be called deleterious by at least 7 functional prediction tools and variants not called by both of the variant caller GATK & Mutect, left with total 99 variants in 26 genes.

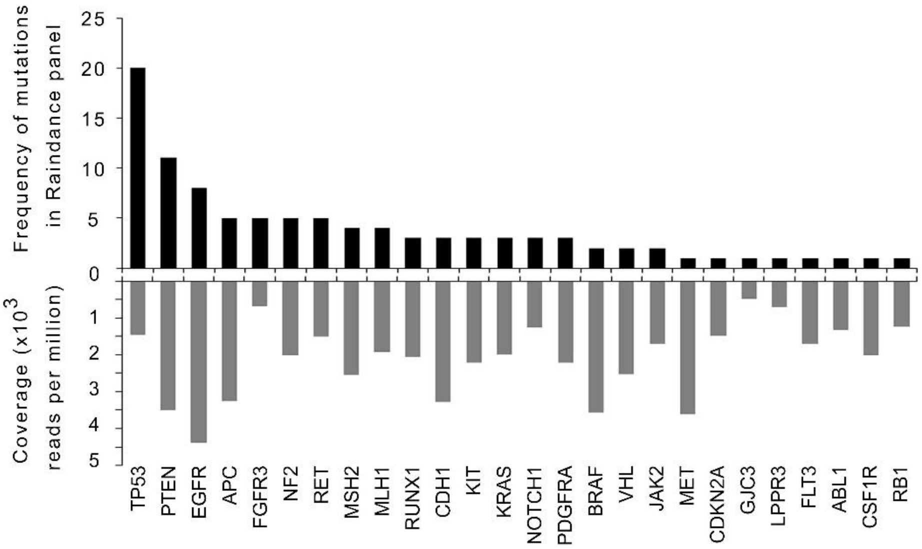

Supplementary Figure-S4: Recurrently mutated genes in lung adenocarcinoma. Recurrently mutated genes identified in 45 lung adenocarcinomas using microfluidics-based high-throughput targeted sequencing of 158 cancer-related genes are shown. Top bar plot represents a percent frequency of mutations in RainDance panel sequencing and bottom bar plot represents sequencing coverage on Illumina platform as reads per million mapped reads.

No.	Gene	Mutation	5XA	5XB	10XA	10XB	15X	Unk
1	ABL1	G250R	■					■
2	APC	L1482F		■				■
3	APC	M1583V	■					
4	APC	P1369S	■					
5	APC	P1420L			■			■
6	APC	R640W	■					
7	BRAF	G464R		■				■
8	BRAF	V471I	■					
9	CDH1	D400N		■			■	■
10	CDH1	P160S	■		■			■
11	CDH1	V685M		■	■		■	
12	CDKN2A	G35E			■			
13	CSF1R	P566L			■			
14	EGFR	ELREA746del	■	■	■		■	■
15	EGFR	G779S		■				
16	EGFR	L858M	■					■
17	EGFR	L858R	■	■			■	■
18	EGFR	R831C	■	■			■	
19	EGFR	S768I		■				■
20	EGFR	V774A		■		■	■	
21	EGFR	V834A	■					
22	FGFR3	C228R	■					
23	FGFR3	G691R	■	■				■
24	FGFR3	P283S		■			■	
25	FGFR3	S249C	■					
26	FGFR3	S679F			■			
27	FLT3	G617E		■			■	■
28	GJC3	E187K		■		■	■	■
29	JAK2	R564Q	■					■
30	JAK2	S591L	■					■
31	KIT	A784T		■				■
32	KIT	G565R		■				■
33	KIT	P551L	■					■
34	KRAS	A59T			■			
35	KRAS	G12V				■		
36	KRAS	P34L		■			■	■
37	LPPR3	A245T		■			■	
38	MET	G1183D		■				■
39	MLH1	A681T		■				■
40	MLH1	E172K		■			■	■
41	MLH1	L658F	■	■				■
42	MLH1	R265C		■				
43	MSH2	C822R			■			■
44	MSH2	D758N			■			
45	MSH2	S676L	■					
46	MSH2	S743L	■	■			■	■
47	NF2	C133Y	■		■			
48	NF2	E372K		■			■	
49	NF2	R196Q				■		■
50	NF2	R341Q	■					
51	NF2	R418C		■			■	
52	NOTCH1	L2510F		■				■
53	NOTCH1	R1594Q	■					■
54	NOTCH1	T2511I		■				■
55	PDGFRA	H570Y	■					
56	PDGFRA	P155L		■				■
57	PDGFRA	P589Q		■			■	
58	PTEN	A126T				■		
59	PTEN	C105Y	■					
60	PTEN	G127R				■		■
61	PTEN	G129E	■		■			■
62	PTEN	G251D	■	■			■	■
63	PTEN	H123Y				■		
64	PTEN	P30L		■			■	■
65	PTEN	P339S		■				■
66	PTEN	R130Q		■				■
67	PTEN	S227F				■		
68	PTEN	V133I	■					■
69	RB1	R798W		■			■	■
70	RET	A756V	■					■
71	RET	R721Q			■			
72	RET	R873W		■			■	
73	RET	R912W		■				■
74	RET	S891L		■				
75	RUNX1	P86S		■			■	
76	RUNX1	R135K		■				■
77	RUNX1	R223H		■			■	■
78	TP53	A138T		■			■	
79	TP53	A276V			■			
80	TP53	A347T		■				■
81	TP53	C141Y	■					
82	TP53	C277F		■			■	
83	TP53	D208N		■			■	
84	TP53	E171G		■			■	
85	TP53	E271K		■			■	
86	TP53	G262V			■			
87	TP53	L130F		■				■
88	TP53	P191H		■			■	
89	TP53	R175H				■		
90	TP53	R273H				■		
91	TP53	R280G	■					■
92	TP53	S269I				■		
93	TP53	T118I		■				
94	TP53	T125M		■	■		■	
95	TP53	T230A		■			■	
96	TP53	T230I		■			■	
97	TP53	Y220N			■			
98	VHL	G144E		■			■	■
99	VHL	V74A	■			■		

Supplementary Figure-S5: List of mutations identified by high-throughput sequencing. List of 99 mutations in 23 genes qualifying after FFPE signature, dbSNP and TMC-SNPdb and functional prioritization filters is shown. Grey box indicates the presence of a mutation in the given pool.

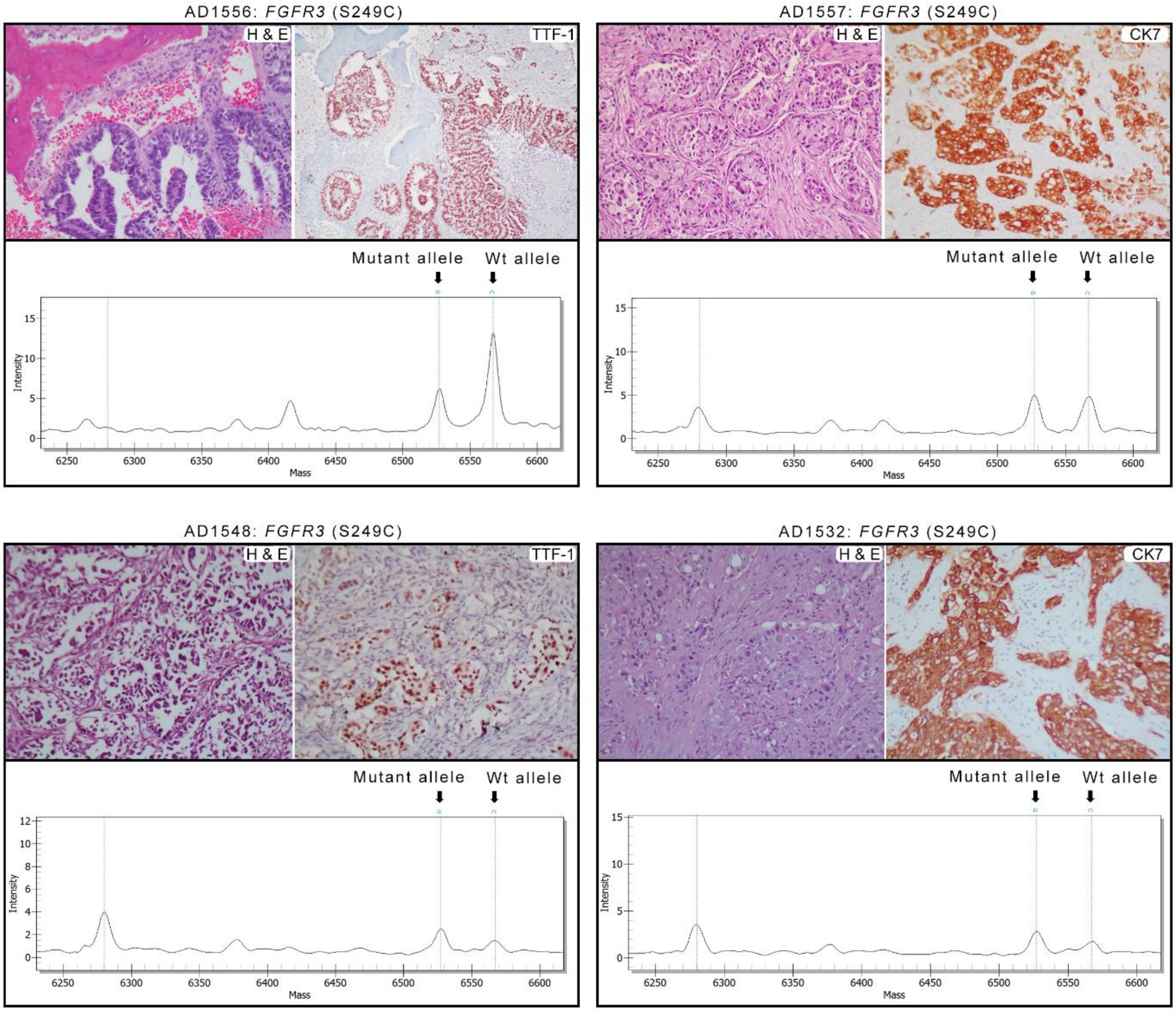

Supplementary Figure-S6: Immunohistochemical and mutational analysis of samples harboring *FGFR3* mutations. Representative images of immunohistochemical (IHC) analysis and spectra of *FGFR3* mutation in lung adenocarcinoma are shown. Upper panel: Hematoxylin and Eosin stain (H & E; on left) showing classical adenocarcinoma morphology. None of the samples showed adenosquamous carcinoma histology. Thyroid transcription factor-1 (TTF-1) or cytokeratin 7 (CK7) positive immunostaining are shown on right. Lower panel: Spectra of *FGFR3* mutations detected by mass-spectrometry are shown. Wild-type allele (Wt) peak and mutant allele peak are indicated with arrows.

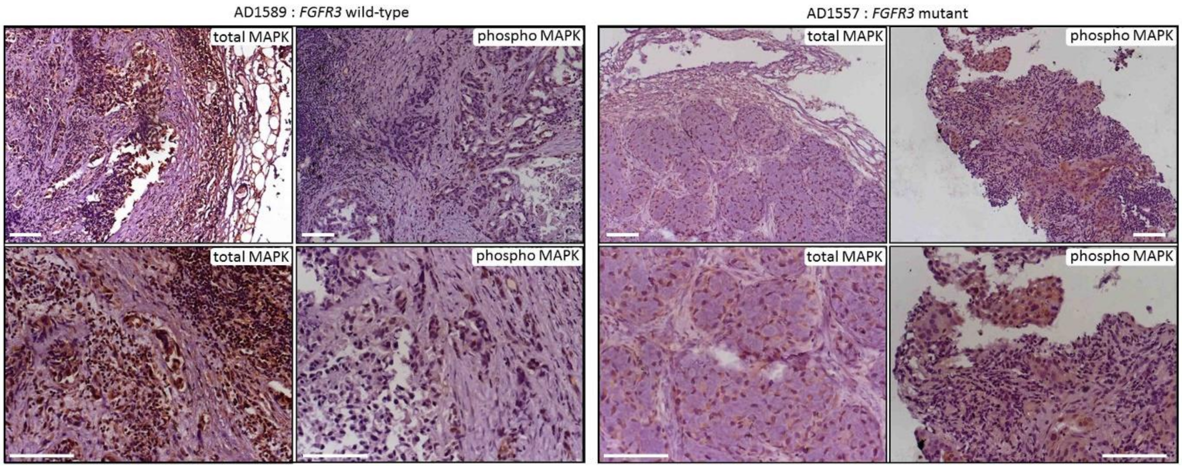

Supplementary Figure-S7: Immuno-histochemical analysis of MAPK activation in lung adenocarcinoma patients with mutant and wild-type *FGFR3*. Representative images of immune-histochemical (IHC) analysis of total- and phospho-MAPK in lung adenocarcinoma patient samples are shown. AD1589 is wild-type for *EGFR*, *KRAS*, *FGFR3*, *EML4-ALK*, *AKT1*, and *ERBB2* while AD1557 is mutant for *FGFR3* and wild-type for *EGFR*, *KRAS*, *EML4-ALK*, *AKT1*, and *ERBB2*. Upper panel: 10x and lower panel: 20x magnification.

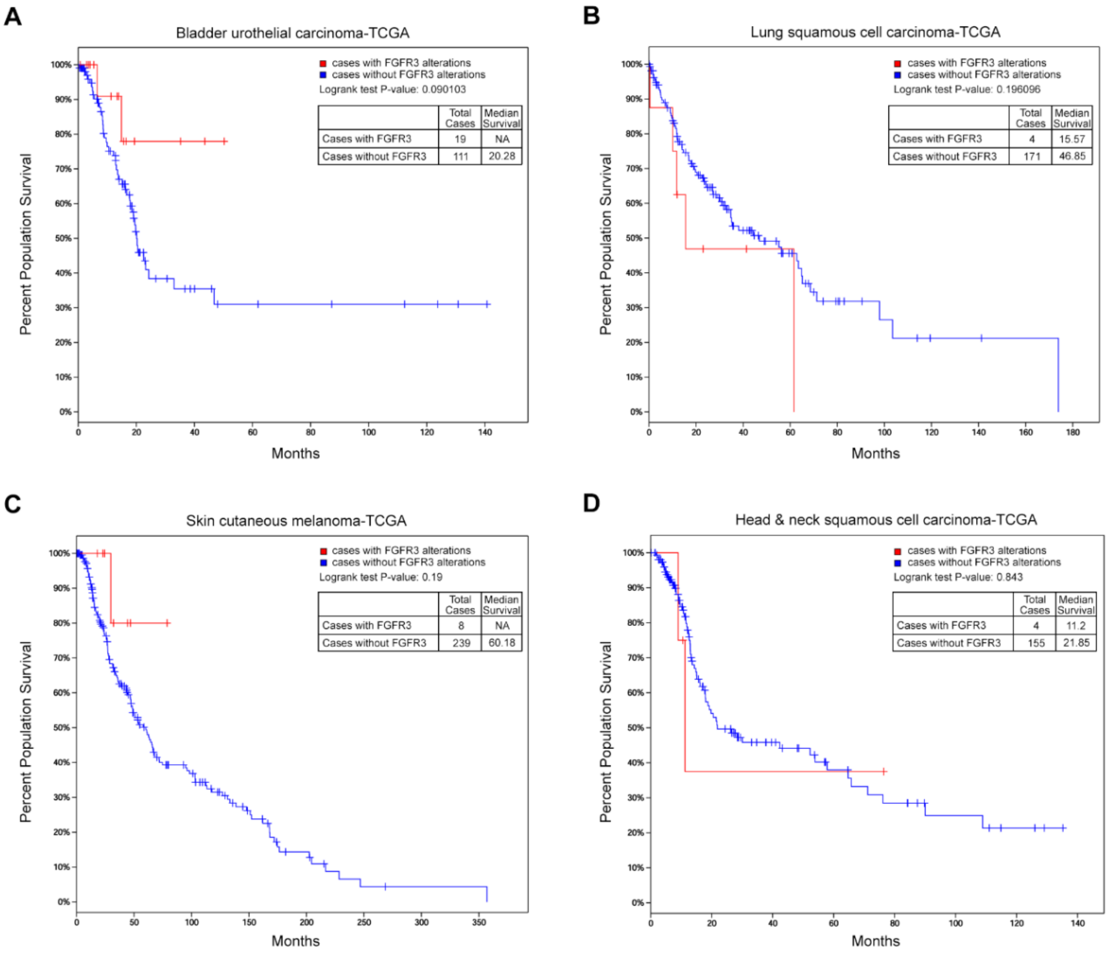

Supplementary Figure-S8: Kaplan-Meier overall survival analysis of patients harboring *FGFR3* mutations using cBioPortal. We queried cBioPortal for *FGFR3* mutant patients in TCGA cohort to compare survival with *FGFR3* wild-type population. Four cancer types in cBioPortal were identified with *FGFR3* mutations and availability of survival information, (a) bladder urothelial carcinoma (b) lung squamous cell carcinoma, (c) skin cutaneous melanoma and (d) head & neck squamous cell carcinoma. Survival curve of *FGFR3* mutant patients (red) and *FGFR3* wild-type patients (blue) is shown.

References

1. Harismendy O, Schwab RB, Bao L et al. Detection of low prevalence somatic mutations in solid tumors with ultra-deep targeted sequencing. Genome Biol 2011; 12: R124.
2. DePristo MA, Banks E, Poplin R et al. A framework for variation discovery and genotyping using next-generation DNA sequencing data. Nat Genet 2011; 43: 491-498.
3. Cibulskis K, Lawrence MS, Carter SL et al. Sensitive detection of somatic point mutations in impure and heterogeneous cancer samples. Nat Biotechnol 2013; 31: 213-219.
4. Wong SQ, Li J, Tan AY et al. Sequence artefacts in a prospective series of formalin-fixed tumours tested for mutations in hotspot regions by massively parallel sequencing. BMC Med Genomics 2014; 7: 23.
5. Upadhyay P, Gardi N, Desai S et al. TMC-SNPdb: an Indian germline variant database derived from whole exome sequences. Database (Oxford) 2016; 2016.
6. Johannessen CM, Boehm JS, Kim SY et al. COT drives resistance to RAF inhibition through MAP kinase pathway reactivation. Nature 2010; 468: 968-972.
7. Chandrani P, Upadhyay P, Iyer P et al. Integrated genomics approach to identify biologically relevant alterations in fewer samples. BMC Genomics 2015; 16: 936.
8. CenterWatch. FDA Approved Drugs for Oncology. In. https://www.centerwatch.com/drug-information/fda-approved-drugs/therapeutic-area/12/oncology: CenterWatch 2016.
9. Guagnano V, Furet P, Spanka C et al. Discovery of 3-(2,6-dichloro-3,5-dimethoxy-phenyl)-1-{6-[4-(4-ethyl-piperazin-1-yl)-phenylamin o]-pyrimidin-4-yl}-1-methyl-urea (NVP-BGJ398), a potent and selective inhibitor of the fibroblast growth factor receptor family of receptor tyrosine kinase. J Med Chem 2011; 54: 7066-7083.
10. Guagnano V, Kauffmann A, Wohrle S et al. FGFR genetic alterations predict for sensitivity to NVP-BGJ398, a selective pan-FGFR inhibitor. Cancer Discov 2012; 2: 1118-1133.
11. Upadhyay P, Nair S, Kaur E et al. Notch pathway activation is essential for maintenance of stem-like cells in early tongue cancer. Oncotarget 2016.
